# Needle-Stick and Sharp Injuries among Hospital Healthcare Workers in Saudi Arabia: A Cross-Sectional Survey

**DOI:** 10.3390/ijerph19106342

**Published:** 2022-05-23

**Authors:** Adil Abalkhail, Russell Kabir, Yousif Mohammed Elmosaad, Ameen S. S. Alwashmi, Fahad A. Alhumaydhi, Thamer Alslamah, Khalid A. Almoammar, Yasir Ahmed Alsalamah, Ilias Mahmud

**Affiliations:** 1 Department of Public Health, College of Public Health and Health Informatics, Qassim University, Al Bukairiyah 52741, Saudi Arabia; abalkhail@qu.edu.sa (A.A.); 4037@qu.edu.sa (T.A.); 2 School of Allied Health, Faculty of Health, Education, Medicine and Social Care, Anglia Ruskin University, Chelmsford CM1 1SQ, Essex, UK; russell.kabir@aru.ac.uk; 3 Department of Public Health, College of Applied Medical Sciences, King Faisal University, Al Hufuf 36362, Saudi Arabia; yelmosaad@kfu.edu.sa; 4 Department of Medical Laboratories, College of Applied Medical Sciences, Qassim University, Buraydah 52571, Saudi Arabia; aswshmy@qu.edu.sa (A.S.S.A.); f.alhumaydhi@qu.edu.sa (F.A.A.); 5 Department of Pediatric Dentistry & Orthodontics, College of Dentistry, King Saud University, Riyadh 11545, Saudi Arabia; kalmoammar@ksu.edu.sa; 6 Department of Surgery, Unaizah College of Medicine and Medical Sciences, Qassim University, Unaizah 56434, Saudi Arabia; y.alsalamah@qu.edu.sa

**Keywords:** needle-stick or sharp injuries, hospital-acquired infection, biological hazards, infection control

## Abstract

Needle-stick or sharp injuries (NSIs) are critical occupational hazards for healthcare workers. Exposure to blood and body fluids through NSIs increases the risk of transmission of blood-borne pathogens among them. The objectives of this study were to estimate the annual incidence of NSIs and investigate the associated factors of NSIs among the healthcare workers in Saudi Arabia. A cross-sectional online survey was conducted between October and November 2021. A total of 361 healthcare workers participated in the survey from all over Saudi Arabia. The one-year incidence of at least one event of NSIs among the healthcare workers is estimated at 22.2% (95% CI: 18.0, 26.8). More than half of the injury events (53.8%) were not reported to the authority by the healthcare workers. Incidence of NSIs was highest among the physicians (36%) and was followed by nurses (34.8%), dentists (29.2%), and medical technologists (21.1%). The odds of NSIs was higher among the healthcare workers aged 26–30 years compared to the 20–25 years age group (OR: 2.51; 95% CI: 1.04, 6.03), as well as among the workers who directly dealt with needles or other sharp objects while working compared to those who did not (OR: 5.9; 95% CI: 2.69, 12.97). The high incidence and low rate of reporting of NSIs highlights the need of education and awareness raising programs targeting healthcare providers with higher risk of injury.

## 1. Background

Needle-stick or sharp injuries (NSIs) are important occupational hazards for healthcare workers, which is often associated with their practice standards [1,2,3]. Exposure to blood and body fluids through NSIs increases the risk of transmission of blood-borne pathogens such as human immunodeficiency virus (HIV), hepatitis B virus (HBV), and hepatitis C virus among healthcare workers [3].

Despite the World Health Organization’s (WHO) guidelines to reduce NSIs in healthcare settings, they continue to occur in every step of sharp devices usage or disposal [4]. Globally, an estimated 32.4–44.5% healthcare workers report at least one event of accidental needle-stick or sharp injury each year [1,2]. In the USA, there are an estimated 385,000 annual incident of NSIs among the hospital healthcare workers [5], while 1,000,000 NSIs cases were reported annually among the hospital healthcare workers in Europe [1].

The risk of injuries to healthcare workers is influenced by several factors, including the type of needle and other sharp objects used, and their safety systems [6]. The risks of NSIs in the healthcare facilities also depends on the number of patients and the precautions the healthcare workers observe while dealing with these patients [6]. The risk of NSIs is high for physicians, nurses, laboratory technicians, and medical waste management workers during screening, diagnosing, treating, and monitoring patients, and during the medical waste management process [7].

A study done in a few governmental hospitals in the Kingdom of Saudi Arabia (KSA) estimated that the annual NSI incidence was 3.2 per 100 occupied beds, and nurses were the most affected job category [4]. A recent study conducted in a hospital in the Medina region estimated the annual incidence of NSIs among healthcare personnel at 32% [8]. Another study by AlDakhil et al. reported that 29.8% of the dental assistants working in private dental clinics in Jeddah, KSA experience at least one event of NSIs since starting their career [9].

Healthcare workers are at risk of getting infected with blood-borne pathogens through NSIs at work. Globally, about 40% of HBV and HCV, and 2.5% of HIV/AIDS cases among the healthcare workers are due to NSIs [3]. More than 90% of these infections occur in healthcare settings in low-income countries where adherence to standard precautions is poor [3].

Despite the high incidence and risk of adverse health consequences of the NSIs [10], there is a marked underreporting of NSI incidents by the healthcare workers [9,11,12]. A study in private clinics in Jeddah, KSA found that more than half (63%) of the dental healthcare workers experiencing NSIs do not report their injuries to the authority [9].

In Saudi Arabia, few studies were conducted regarding NSIs among health care workers. To add to the growing evidence on NSIs and their risk factors, our study focused on healthcare workers at hospitals and dental clinics in Saudi Arabia, and investigated the incidence and associated factors of NSIs—demographic characteristics, nature of work, exposure to training programs, work experience, and educational attainment.

## 2. Methods

### 2.1. Study Design and Sampling

We did a cross-sectional survey of the healthcare workers in hospitals and dental clinics in the KSA. The country has an estimated 350 thousand healthcare workers [13]. We conducted the survey online, using the Google Form, between October and November 2021. The online survey link was disseminated to the healthcare workers throughout the KSA through different professional and social networks, such as emails and professional WhatsApp groups. We indicated our study objectives and title clearly on the front page of the online survey form, and the participants were requested to avoid multi-registration. The participants who provided informed consent were allowed to visit the subsequent pages to participate in the survey. A total of 366 healthcare workers from public and private healthcare institutions participated in this online survey. We dropped five participants from analyses because of incomplete information. Table 1 presents the characteristics of the participants.

### 2.2. The Instrument

We used a structured questionnaire. The structured questionnaire collected information on the participants’ socio-demographic variables, work characteristics, and NSIs events in the past twelve months. Respondents only recorded their last injury event within the past twelve months. Questions on NSI events were adapted from the EPINet Needlestick and Sharp Object Injury Report form [14].

### 2.3. Variables

The main outcome variable was NSIs events in the past twelve months. The other outcome variables included reporting status of the injury event to the hospital authority, activities led to the injury, place of injury, type of device which caused the injury, contamination status of the device caused the injury, and severity of the injury. Our explanatory or predictor variables included job title, age, gender, years of professional experience, training on NSIs prevention, and whether the job requires them to directly deal with sharp objects.

### 2.4. Analysis

SPSS version 20 (IBM, Armonk, NY, USA) was used for data analysis. We did descriptive analysis of the sociodemographic variables and the outcome variables including the NSIs events. We reported frequency and percentages for each of the categorical variables. We reported number and proportions of the healthcare workers who had injuries in the past twelve months, and proportion and number of the healthcare workers who reported their injury event to the authority.

We did simple and multivariable logistic regression analyses to investigate the predictors of NSIs. For logistic regression analyses, we reported odds ratio (OR) with 95% confidence interval (CI). In addition, we reported corresponding *p* values. A *p* value of <0.05 was considered statistically significant.

## 3. Results

A total of 366 healthcare workers participated in the study (5 of them were excluded from analyses because of incomplete responses). Our participants included medical technologist (24.9%), nurses (19.1%), medical, dental or health science students (16.1%), physicians (13.9%), volunteers (10.8%), dentists (6.6%), pharmacists (6.1%), and housekeeping staff (2.5%). The participants were predominantly female (70.6%), Saudi (83.1%), and 41.6% of them belonged to the younger age group (20–25 years). Among the participants, 55.1% had professional experience up to 5 years, 56.8% were dealing with sharp materials at work, but only 45.2% received training on dealing with sharp objects (see Table 1).

A total of 80 participants, that is 22.2% (95% CI: 18.0–26.8%), experienced at least one event of NSIs in the past year. The rate was 28.3% among the males and 19.6% among the females. Incidence among the physicians was 36%; nurses 34.8%; dentists 29.2%; medical technologists 21.1%; pharmacist 13.6%, and housekeeping staff 11.1%. Non-Saudi (27.9%) healthcare workers are estimated to have higher incidence than Saudis (21%). We observed higher annual incidence of at least on NSI events among the older age groups (30% and 29.6% respectively in 30–39 years and >39 years age group) compared to the younger age groups (20–29 years, 17.1%) (see Table 2).

Table 3 shows the unadjusted and adjusted odds ratios and their 95% confidence intervals (CIs) for the predictors of NSIs among the hospital healthcare workers. We observed that the odds of NSIs were 2.5 (95% CI: 1.04, 6.03) times higher in 26–30 years age group compared to the 20–25 years age group after adjusting for the effect of other socio-demographic and profession related variables. However, no significant differences in the odds of NSIs were observed between 20–25 years and other older age groups.

Our multivariable logistic regression analysis also suggests that dealing with sharp objects at work is significantly associated with NSIs experience. The odds of NSIs among the workers who deal with sharp objects at work was 5.9 (95% CI: 2.69–12.97) times the odds of NSIs among the healthcare workers who do not deal with sharp objects at work.

Physicians, nurses, dentists, and medical technologist were significantly associated with higher odds of NSIs compared to volunteer healthcare workers in unadjusted analyses but in the adjusted model, no such association was evident. No significant differences in the odds of NSIs were observed between gender and nationality groups in both the unadjusted and adjusted models.

The area of the hospital where most of the NSIs took place were the emergency unit (33.8%), followed by the laboratory (22.5%), surgery unit (17.5%), vaccination center (11.3%), dental clinic (7.5%), and the ICU (2.5%).

With regard to contamination status of the sharp item, about 34% of the injured healthcare workers were injured with a sharp item contaminated with blood, 16.3% were injured with a sharp item contaminated with other biohazards, while 23.8% were injured with uncontaminated sharp items, and 26.3% were injured with a sharp item with an unknown contamination status.

Regarding the device caused the injury, we found that the most common devices leading to NSIs were needles (53.8%), followed by blades (22.5%), glassware (10%), plastic ware (6.3%), and scissors (2.5%).

Most injuries occurred during using a device (47.5%), followed by recapping a used needle (16.3%), and dissembling a device/equipment for cleaning/sterilization (11.3%), and 3.8% NSIs occurred while disposing of a used device. About 8.8% of the healthcare workers received injures while doing other activities.

The majority (70.0%) of the injured healthcare workers reported mild injuries, while 22.5% reported moderate injuries (skin puncture/cut, some bleeding), and only 7.5% reported severe (deep puncture/cut or abundant bleeding) injuries. We found that more than half of the healthcare workers who experienced at least one NSIs event in the preceding year (53.8%) did not report their last injury event to the authority (Table 4).

## 4. Discussion

Needle-stick or sharp injuries (NSIs) are a prominent occupational threat for healthcare workers [1,2]. It is essential to identify the rate of NSIs and their associated factors among hospital healthcare workers in the KSA. Our findings showed that 22.2% of the participants experienced at least one event of NSIs in the preceding year. This finding is almost similar to that reported in Turkey (20.7%) [15], but slightly lower than those reported in Germany (28.7%) [16], and China (27.5%) [17]. Similar studies conducted in other parts of KSA, Jeddah [9] and Medina [8], reported slightly higher incidence—29.8% and 32%, respectively. Another study conducted in Dammam reported a very low rate (8.4%) when compared to our study’s findings [4]. Conversely, much higher incidence was reported in South Korea (70.4%) [18], Ethiopia (60.2%) [19], and Iran (42.5%) [20]. It is possible that greater compliance with infection control procedures, workplace safety awareness, and available resources are associated with lower risk of NSIs in the KSA. Nevertheless, the annual incidence (22.2%) reported by our study is alarming.

Regarding the healthcare workers exposed to NSIs while performing clinical activities, our findings showed that the most common injured groups were the physicians (36%), nurses (34.8%), dentists (29.2%), and medical technologists (21.1%). Albeladi et al. [8] reported slightly different rate among nurses (38.6%) and physicians (30.4%) and markedly different rate among laboratory technicians (13.9%) in emergency departments of hospitals in Median, KSA. These differences could be because of the small number of participants in each occupation group and doing studies in different settings. Further studies targeting individual occupation groups with larger sample sizes are recommended. In relation to the rate of NSIs among dentists, our estimate (29.2%) is less than the neighboring countries such as Jordan (66.5%) [21] and the United Arab Emirates (42%) [22], but similar to the rate (29.8%) among the dental care assistants in Jeddah, KSA [9]. The lower prevalence of NSIs among nurses, dentists, and laboratory technicians in the KSA might be because of the provision of workplace safety, adequate clinical experience, and continuous training programs on infection control.

Our findings illustrated that physicians, nurses, dentists, and laboratory technicians reported higher incidence of NSIs compared to pharmacists and housekeeping staff, trainee students and volunteers. This could be because they directly deal with sharp objects at work, as we also found that dealing with sharp objects at work is significantly associated with NSIs experience. Age is also a significant predictor of the risk of NSIs; it was found that participants belonged to the 26–30 years age group were significantly associated with higher odds of NSIs compared to the younger group. We found lack of significant association between gender, years of experience and attending training on NSIs prevention and NSIs events among the healthcare workers in the KSA. This result is in contrast to previous studies conducted in Iran, Portuguese and Sub-Saharan Africa [23,24,25]. Not finding any beneficial effect of training perhaps highlights the needs of looking at the content and delivery methods of such training programs.

In our study, the area of the hospital where most of the NSIs took place was the emergency unit; this finding was congruent with studies conducted in KSA and India [4,26]. The other most common place the NSIs took place were laboratories, and this finding was similar to a study conducted in Southern Ethiopia [27]. Moreover, our findings showed that the surgery unit was also associated with NSIs. This result is similar to other studies that show surgery units are the most common place of NSIs occurrence [28]. Higher incidence of NSIs in these areas can be explained by the nature of the work and the medical activities carried out in these areas.

We noted that different devices led to NSIs in hospitals in the KSA. The most common devices were needles followed by blades, glassware, plastic ware, and scissors. The findings of our study especially related to the fact that needles caused majority of the injury were consistent with the results of other studies conducted in Portuguese [24], and India [29].

Our study suggested that the most common activity leading to NSIs was while using the device and recapping used needles. This was consistent with previous investigations from Nigeria [30], Southern Ethiopia [27], Taiwan [31], and KSA [9]. The other activities leading to NSI were dissembling a device/equipment for cleaning/sterilization.

In our study, 53.8% of the injured participants did not notify the authority when they had NSIs. High underreporting (63%) of the NSI events is also reported by a study conducted in Jeddah, KSA [9]. This indicated that there is a marked underreporting of NSIs events by healthcare workers in the KSA, similar to other countries [12]. A study in South Korea reported that more than two thirds of the hospital healthcare workers experiencing NSIs did not report those injuries to the authority [12]. This might be due to the assumption that no blood-borne pathogens existed in the source patient, lack of adherence to standard infection control precautions, or lack of awareness about the reporting procedure. This lack of reporting could also be due the fact that healthcare workers, in majority of the cases, do not perceive the injury as severe. For example, regarding the severity of injuries among hospital healthcare workers, our results showed that most of the injured hospital healthcare workers (70%) reported having mild injuries with little or no bleeding.

We conducted a cross-sectional survey to estimate the one-year incidence of NSIs among healthcare workers in the KSA. This design is subject to recall bias, and we cannot rule out the possibility that individuals who experienced the NSIs participated more than the ones who did not had injuries. Furthermore, we recruited participants through open online invitation. Hence, we could not select the participants randomly. This sampling technique might have negative influence on the generalizability of our findings. Nevertheless, this study provided important insight into this critical occupational health issue. Future studies should employ a cohort design targeting specific occupation group.

## 5. Conclusions

We reported a high annual incidence (22.2%) of at least one NSI event among healthcare workers in Saudi Arabia, and a low rate of reporting (46.3%) of such injury events. The most common affected groups are the physicians, followed by nurses, dentists, and medical technologists. In addition, our study highlighted that NSIs risk is higher among healthcare workers belonged to 26–30 years, and most cases of NSIs take place in emergency units, laboratories, and surgery units. The most common activities leading to NSIs were using the device and recapping used needles. An education program should be designed targeting healthcare providers with higher risk. Moreover, a unified needle-stick and sharp injuries policy is warranted covering safe work practices, safe disposal of needles and other sharp objects, procedures in the event of an injury, procedures for reporting needle-stick or sharp injuries, and training to avoid these injuries.

## Figures and Tables

**Table 1:** Characteristics of the hospital healthcare workers.

Variables	Frequency	Percent
**Job title**		
Physician	50	13.9
Dentist	24	6.6
Nurse	69	19.1
Medical technologist	90	24.9
Pharmacist	22	6.1
Housekeeping staff	9	2.5
Student (medical/dental/health science)	58	16.1
Volunteer	39	10.8
**Age groups**		
20–25 years	150	41.6
26–30 years	67	18.6
31–40 years	90	24.9
41–50 years	40	11.1
51–60 years	14	3.9
**Sex**		
Male	106	29.4
Female	255	70.6
**Nationality**		
Saudi	300	83.1
Non-Saudi	61	16.9
**Professional experience**		
Up to 5 years	199	55.1
6–10 years	71	19.7
11–20 years	63	17.5
More than 20 years	28	7.8
**Received training on dealing with sharp objects**		
No	198	54.8
Yes	163	45.2
**Dealing with sharp objects at work**		
No	156	43.2
Yes	205	56.8

**Table 2:** One-year incidence of needle-stick or sharp injuries among hospital healthcare workers in Saudi Arabia.

Variables	Incidence (One Year)
	**Percent (95% CI)**
**Total**	22.2 (18.0–26.8)
**Gender**	
Male	28.3 (20.0–37.9)
Female	19.6 (14.9–25.0)
**Age group**	
20–29 years	17.1 (12.3–22.7)
30–39 years	30.0 (20.8–40.6)
40 years or more	29.6 (18.0–43.6)
**Nationality**	
Saudi	21.0 (16.5–26.1)
Non-Saudi	27.9 (17.1–40.8)
**Job title**	
Physician	36.0 (22.9–50.8)
Nurse	34.8 (23.7–47.2)
Dentist	29.2 (12.6–51.1)
Medical technologist	21.1 (13.2–31.0)
Pharmacist	13.6 (2.9–34.9)
Housekeeping staff	11.1 (0.3–48.2)
Student	12.1 (5.0–23.3)
Volunteer	2.6 (0.1–13.5)

**Table 3:** Predictors of needle-stick or sharp injuries among hospital healthcare workers in Saudi Arabia.

Predictors	Unadjusted Model		Adjusted Model	
	OR (95% CI)	*p*-Value	OR (95% CI)	*p*-Value
**Gender**				
Female	1		1	
Male	1.62 (0.96, 2.73)	0.072	1.17 (0.61, 2.27)	0.638
**Age**				
20–25	1		1	
26–30	2.90 (1.41, 5.99)	0.004	2.51 (1.04, 6.03)	0.040
31–40	3.14 (1.61, 6.13)	0.001	1.43 (0.47, 4.40)	0.531
41–50	2.78 (1.19, 6.51)	0.018	2.71 (0.72, 10.13)	0.139
51–60	4.07 (1.23, 13.51)	0.022	3.48 (0.61, 19.93)	0.162
**Nationality**				
Non-Saudi	1		1	
Saudi	0.69 (0.37, 1.29)	0.241	1.11 (0.49, 2.51)	0.811
**Job title**				
Volunteer	1		1	
Physician	21.37 (2.70, 169.03)	0.004	6.77 (0.74, 62.32)	0.091
Dentist	15.65 (1.78, 137.31)	0.013	5.53 (0.56, 54.48)	0.143
Nurse	20.27 (2.62, 156.87)	0.004	7.20 (0.82, 63.08)	0.075
Medical technologist	10.17 (1.31, 78.92)	0.027	3.78 (0.42, 33.81)	0.235
Pharmacist	6.00 (0.58, 61.62)	0.132	7.11 (0.61, 83.41)	0.118
Housekeeping staff	4.75 (0.27, 84.17)	0.288	1.32 (0.06, 29.72)	0.859
Student	5.22 (0.62, 44.20)	0.130	5.92 (0.61, 57.54)	0.125
**Professional experience**				
Up to 5 years	1		1	
6–10 years	3.13 (1.69, 5.80)	0.000	1.99 (0.77, 5.15)	0.155
11–20 years	1.84 (0.93, 3.66)	0.080	0.79 (0.26, 2.40)	0.673
More than 20 years	1.81 (0.71, 4.61)	0.216	0.53 (0.12, 2.28)	0.395
**Received training on dealing with sharp objects**				
No	1		1	
Yes	2.32 (1.39, 3.85)	0.001	1.39 (0.79, 2.47)	0.253
**Dealing with sharp objects at work**				
No	1		1	
Yes	6.69 (3.40, 13.17)	0.000	5.90 (2.69, 12.97)	<0.001

**Table 4:** Description of the needle-stick or sharp injury event.

Variables	Frequency	Percent
**Place of injury**		
Dental clinic	6	7.5
Emergency unit	27	33.8
ICU	2	2.5
Laboratory	18	22.5
Surgery unit	14	17.5
Vaccination centre	9	11.3
**Contamination status of the sharp item**		
Contaminated with blood	27	33.8
Contaminated with other biohazards	13	16.3
Uncontaminated	19	23.8
Unknown	21	26.3
**Device caused the injury**		
Needle	43	53.8
Blade	18	22.5
Glassware	8	10.0
Plasticware	5	6.3
Pipette	3	3.8
Scissors	2	2.5
Not sure	1	1.3
**The activity that led to the injury**		
While assembling or preparing the device to use	8	10.0
While using the device	38	47.5
While removing a disposal container or trash bag	2	2.5
While recapping a used needle	13	16.3
While dissembling a device/equipment for cleaning/sterilization	9	11.3
While disposing off a used device	3	3.8
Other	7	8.8
**Severity of the injury**		
Mild (superficial, little or no bleeding)	56	70.0
Moderate (skin puncture/cut, some bleeding)	18	22.5
Sever (deep puncture/cut or abundant bleeding)	6	7.5
**Notified the hospital authority about the injury**		
No	43	53.8
Yes	37	46.3

## Data Availability

Data used in this study are available from the corresponding author on reasonable request.
